# TBSure: an ESGMYC survey of pediatric TB management practices in high-income countries

**DOI:** 10.3389/fpubh.2026.1829612

**Published:** 2026-05-19

**Authors:** Anita Uka, Frédéric Méchaï, Daniela Maria Cirillo, Lorenzo Guglielmetti, Yousra Kherabi, Daria Podlekareva, Pierre Alex Crisinel, Onya Opota, on behalf of the ESGMYC group

**Affiliations:** 1 Service of Pediatrics, Department of Women–Mother–Child, Lausanne University Hospital, Lausanne, Switzerland; 2 Department of Infectious Diseases, Avicenne Hospital, Hôpitaux Universitaires Paris Seine-Saint-Denis, Bobigny, France; 3 IAME, INSERM UMR 1137, INSERM, Université Paris Cité, Paris, France; 4 Division of Immunology, Transplant and Infectious Diseases, IRCCS San Raffaele Scientific Institute, Milan, Italy; 5 Department of Infectious, Tropical Diseases and Microbiology, IRCCS Sacro Cuore Don Calabria Hospital, Negrar di Valpolicella, Verona, Italy; 6 Infectious and Tropical Diseases Department, Bichat-Claude Bernard Hospital, Assistance Publique–Hôpitaux de Paris, Paris, France; 7 International Reference Laboratory of Mycobacteriology (IRLM), Statens Serum Institut, Copenhagen, Denmark; 8 Department of Respiratory Medicine and Infectious Diseases, Copenhagen University Hospital – Bispebjerg, Copenhagen, Denmark; 9 Faculty of Medical Science, University of Copenhagen, Copenhagen, Denmark; 10 Unit of Pediatric Infectious Diseases and Vaccinology, Service of Pediatrics, Department Women–Mother–Child, Lausanne University Hospital and University of Lausanne, Lausanne, Switzerland; 11 Institute of Microbiology, University of Lausanne University Hospital, Lausanne, Switzerland

**Keywords:** guideline adherence, molecular diagnostics, pediatric tuberculosis, practice variation, tuberculosis diagnosis

## Abstract

**Introduction:**

Tuberculosis (TB) remains a leading cause of pediatric morbidity and mortality worldwide, with diagnostic and therapeutic challenges continuing to affect outcomes, including in high-income settings. The TBSure study investigated current practices in pediatric TB diagnosis and management across multiple countries, aiming to identify variations, strengths, and gaps in real-world care and to inform evidence-based recommendations.

**Methods:**

We conducted a cross-sectional, structured survey distributed to healthcare professionals involved in pediatric TB care through a professional network. The questionnaire assessed diagnostic approaches, specimen selection, use of microbiological and molecular tests, treatment regimens for active and latent TB, monitoring practices, and adherence to international (WHO 2022, ERS/ECDC 2017 and IJTLD 2023) guidelines. Descriptive analyses were performed to summarize practices and identify variability across settings.

**Results:**

A total of 60 participants from 17 countries responded, predominantly from Europe. In children unable to expectorate, early-morning gastric aspirates were used by 47% of respondents, induced sputum or gastric aspirate depending on age by 29%, and induced sputum alone by 18%. Smear microscopy and culture were systematically performed by 86 and 63% of respondents, respectively. Rapid molecular testing was routinely performed by 80%. Culture was systematically requested following negative nucleic acid amplification test results in 88% of cases. Molecular drug-resistance testing on initial samples was commonly requested, most frequently for rifampicin resistance alone (47%) or combined rifampicin and isoniazid resistance (41%). Treatment practices for presumed drug-susceptible tuberculosis were uniform across respondents, with universal use of the standard 6-month regimen. However, implementation of the 4-month regimen for non-severe disease was inconsistent, being reported by only 47% of respondents. Fluoroquinolones were used as first-line therapy in cases of suspected isoniazid resistance by 49% of respondents and in tuberculous meningitis by 33%. Baseline blood testing (92%), toxicity monitoring (67%), HIV testing (86%), and post-discharge directly observed therapy (83%) were widely implemented.

**Conclusion:**

Pediatric TB practices are generally aligned with guidelines, with widespread use of rapid molecular testing and standardized treatment regimens, but substantial heterogeneity persists for diagnostic approaches and isolation measures. Strengthening implementation of evolving international recommendations may improve early diagnosis, treatment consistency, and outcomes for children affected by tuberculosis.

## 1 Introduction

Pediatric tuberculosis (TB) remains a public health challenge in Europe, where it represents a small but persistent proportion of the overall TB burden. According to European surveillance data in 2023, children under 15 years of age account for 4.5% of notified TB cases in the EU/EEA, with higher proportions observed among migrants and vulnerable populations, suggesting ongoing transmission and delayed case detection in some settings (1).

In Europe, TB notifications increased over three consecutive years between 2021 and 2023, reflecting ongoing transmission despite overall low incidence levels in many high-income settings (1). Up to 21% of the world’s multidrug-resistant TB burden is estimated to occur in the WHO European Region, posing a substantial challenge for treatment and highlighting the imperative for effective pediatric TB care (1). Despite relatively low incidence of active TB in much of Europe, a substantial reservoir of latent TB infection persists in the region. Modeling estimates suggest that approximately 14% of the European population had latent TB infection in 2014, with about 0.3% of the population having been infected within the prior 2 years and therefore at higher immediate risk of progression to active disease (2). WHO and ECDC strategic plans explicitly support scaling up prevention, early diagnosis, and access to quality treatment to accelerate progress toward End TB Strategy targets (3, 4).

Several international and European initiatives have aimed to standardize TB management. WHO and other expert groups have updated diagnostic algorithms and treatment recommendations, including wider implementation of rapid molecular tests and clearer guidance on pediatric sample collection (5–7). Recent high-quality evidence has demonstrated that shorter treatment regimens are effective for non-severe drug-susceptible pediatric TB, with the SHINE trial showing non-inferiority of a four-month regimen compared with standard six-month therapy (8, 9).

Despite these advances, practice variation persists across high-income countries. A multicountry survey by Méchaï et al. demonstrated substantial heterogeneity in adult TB management practices in high-income countries (10). Comparable data focusing specifically on pediatric TB are scarce, despite important differences between adult and pediatric disease and the emergence of new evidence relevant to children.

The aim of the current study is to evaluate and compare current practices in the diagnosis, treatment, and monitoring of pediatric TB across multiple countries, using a methodology analogous to that applied in adult populations. By identifying heterogeneity in pediatric TB management and assessing the uptake of recent recommendations, including shorter treatment regimens for non-severe disease, this study seeks to complement existing adult data and inform the development of more harmonized, evidence-based pediatric TB care in high-income countries.

## 2 Materials and methods

A cross-sectional, anonymous survey was conducted using SurveyMonkey® and distributed via the European Society of Clinical Microbiology and Infectious Diseases Study Group on Mycobacterial Infections (ESGMYC) network between January 2024 and November 2025 among healthcare professionals involved in pediatric TB care, defined as any professional who has managed a confirmed or suspected case of TB disease in a patient under 18 years of age within the past 2 years. This study was conducted as an anonymous survey of healthcare professionals and did not involve any patient data or identifiable information. The survey was distributed by email to ESGMYC members, with reminder emails included in the newsletters of the study group. Participation was open to healthcare professionals involved in pediatric TB care beyond ESGMYC membership. Multiple respondents from the same institution were allowed, as healthcare professionals may have complementary roles and perspectives. The questionnaire (Supplementary material) included 51 questions across six domains: demographics, TB screening, diagnostic approaches, microbiological and molecular tests, infection prevention and control, and management of active and latent TB. Most questions were multiple-choice questions with a few free-text fields that allowed respondents to specify answers. Eligible participants were clinicians and laboratory specialists involved in the management of children (aged 0 to 18 years) with suspected pulmonary or extrapulmonary TB in high-income countries, primarily in Europe. Clarification on ethical responsibility was sought from the local ethic committee (Commission Cantonale d’Ethique de la Recherche sur l’Etre Humain, Lausanne, Switzerland), which determined that this survey did not require formal ethics approval. Descriptive statistical analyses were conducted, with categorical variables summarized as percentages, using R software and Microsoft Excel®. The map was created with R software. Survey findings were compared with WHO (2022), ERS/ECDC (2017), and IJTLD (2023) pediatric tuberculosis recommendations across diagnostic, infection control, and treatment domains, summarized in comparative tables (Tables 1–4).

**Table 1:** Diagnosis of pediatric tuberculosis disease.

Diagnostic practices	Survey findings	WHO module 5 (2022)	ERS/ECDC statement (2017)	IJTLD clinical standards (2023)
Types of specimens	Early-morning gastric aspirate (47%) most requested; induced sputum or gastric aspirate depending on age (29%); sputum only (18%); CSF, tissue biopsies, stool specimens less used	Gastric aspirate or induced sputum; NAAT can also be done on CSF, tissue, stool if indicated	Sputum specimens for microscopy and NAAT; at least one early-morning sample when possible	Gastric aspirate, induced sputum, stool or nasopharyngeal aspirate
Culture	63% perform culture routinely; 88**%** perform culture if NAAT negative	Culture recommended for bacteriologically confirmed cases; culture-based DST performed on positive isolates	Specimens sent for liquid culture and, if positive, for culture-based DST	Culture and DST recommended to confirm diagnosis and guide treatment decisions
Timing and indication of NAAT	80% perform NAAT routinely; 12% only if smear positive	NAAT should be used as initial diagnostic test	NAAT on at least one specimen for TB and drug resistance	NAAT with rapid detection of TB and rifampicin resistance can be used
Number of specimens to collect	41% perform NAAT on up to 3 respiratory samples when initial tests negative	Two specimens for microscopy; one specimen for NAAT; repeat testing if initial NAAT negative and pretest probability ≥5%	At least two sputum specimens for microscopy and one for NAAT	Combining samples improves sensitivity
LF-LAM	6% use LF-LAM to assist in the diagnosis of TB for HIV-positive patients	LF-LAM may be used to assist diagnosis in HIV-positive children, particularly in those who are seriously ill or immunocompromised	LF-LAM mentioned as an adjunctive diagnostic tool in HIV-infected patients	LF-LAM mentioned for children with advanced immunosuppression
Drug resistance detection	NAAT for rifampicin resistance alone in 47%; NAAT for combined rifampicin and isoniazid resistance in 41%; not requested or uncertain in 12%	NAAT for rifampicin resistance at initial diagnosis; additional testing for isoniazid resistance based on bacteriological confirmation, risk factors, and test availability	Early detection of drug resistance emphasized; NAAT recommended where available	NAAT provides rapid detection of rifampicin resistance; Xpert MTB/XDR when available; culture-based DST still required for confirmation
Management of negative microbiology	Treatment initiated despite negative microbiology in 73% in case of strong clinical suspicion; 18% based on severity; 6% awaited culture confirmation	Clinical diagnosis acceptable when microbiological confirmation is not achieved	Clinical diagnosis acceptable when microbiological confirmation is not achieved	Clinical diagnosis acceptable when microbiological confirmation is not achieved

CSF, cerebrospinal fluid; NAAT, nucleic acid amplification test; TB, tuberculosis; DST, drug susceptibility testing; LF-LAM, lateral flow urine lipoarabinomannan assay; HIV, human immunodeficiency virus; Xpert MTB/XDR, molecular assay detecting Mycobacterium tuberculosis and resistance to rifampicin, isoniazid, fluoroquinolones, and second-line injectables.

**Table 2:** Treatment of active tuberculosis.

Treatment scheme	Survey findings	WHO module 5 (2022)	ERS/ECDC statement (2017)	IJTLD clinical standards (2023)
Standard treatment regimen	97% use 2HRZ(E)/4HR regimen for presumed drug-susceptible TB	2HRZ(E)/4HR regimen used when criteria for the 4-month regimen are not met	2HRZE/4HR regimen for drug-susceptible TB	2HRZ(E)/4HR regimen used when criteria for the 4-month regimen are not met
Shortened regimens (4-month)	46% use 4-month regimen for non-severe TB (Turkova criteria)	4-month regimen for eligible children with non-severe TB (Turkova criteria)	NA	4-month regimen for eligible children with non-severe TB (Turkova criteria)
TB meningitis	67% use 2RHZE/10HR; 33% add or replaced ethambutol by fluoroquinolone	2RHZE/10RH recommended; 6-month RHZEth intensive regimen may be used in selected children without drug resistance, excluding HIV-positive patients	NA	2RHZE/10RH recommended; 6-month RHZEth intensive regimen may be used in selected children without drug resistance, excluding HIV-positive patients; ethambutol might be replaced by fluoroquinolones
Adjunctive cortico-steroids	Corticosteroids used by 94%; most commonly for 4 weeks	Corticosteroids tapered over 6–8 weeks recommended	Refer to WHO	Corticosteroids given for 4 weeks then tapered over 2–4 weeks recommended
Pyridoxine supplementation	Routinely prescribed with isoniazid in 47%; selectively in malnutrition, exclusive breastfed, or comorbidities	Recommended for all pregnant/breastfeeding individuals, those with HIV, malnutrition, alcohol use, diabetes, chronic liver or kidney disease	NA	Recommended for children with HIV or malnourished, all infants and adolescents (especially pregnant), and those receiving high-dose isoniazid
Management of MDR-TB	Use of fluoroquinolones as first-line for suspected isoniazid-resistant TB in 49%	MDR-TB managed according to DST; 6REZLfx for confirmed rifampicin-susceptible, isoniazid-resistant TB	MDR-TB managed by specialist center based on DST; Minimum of 5 anti-TB drug for at least 20 months (including 8 months intensive phase)	NA

DST, drug susceptibility testing; H, isoniazid; R, rifampicin; Z, pyrazinamide; E, ethambutol; RHZE, combination of rifampicin, isoniazid, pyrazinamide, and ethambutol; 2HRZ(E)/4HR, standard 6-month first-line TB regimen (2-month intensive phase, 4-month continuation phase); 2RHZE/10HR, 12-month regimen including 2 months of rifampicin, isoniazid, pyrazinamide and ethambutol followed by 10 months of rifampicin and isoniazid; 6REZLfx, a 6-month regimen with rifampicin, ethambutol, pyrazinamide and levofloxacin; MDR-TB, multidrug-resistant tuberculosis; NA, not applicable.

**Table 3:** Monitoring of treatment and isolation practices.

Monitoring and isolation practices	Survey findings	WHO module 5 (2022)	ERS/ECDC statement (2017)	IJTLD clinical standards (2023)
Monitoring of treatment response	Culture performed at multiple time points: 2 weeks (57%), 1 month (26%), 2 months (34%), 3 months (6%), 4 months (6%), end of treatment (14%); 14% no post-treatment culture; baseline blood testing 92%, systematic drug level monitoring uncommon (8%)	Monitoring for treatment response using clinical assessments, weight/height tracking; smear/culture for bacteriologically confirmed cases; biochemical tests according to drug	Pulmonary TB: smear + culture at end of initial phase (2 months); if positive, additional DST and NAAT for drug resistance Extrapulmonary/children unable to expectorate: clinical assessment MDR-TB: monthly smear/culture where feasible	Regular clinical follow-up including symptoms, weight/height; microbiological monitoring guided by clinical course or for severe disease
Toxicity monitoring	68% routine laboratory toxicity monitoring; others only if symptomatic	Routine clinical and laboratory monitoring to detect drug toxicity, with biochemical tests adapted to the specific drug used	NA	Toxicity monitoring recommended for selected case only (pre-existing liver disease or use of other hepatotoxic drugs)
HIV testing in children with suspected or confirmed TB	87% routine HIV testing to all children diagnosed with active TB	Systematic HIV testing recommended, regardless of epidemiological setting	Systematic HIV testing recommended	Systematic HIV testing recommended
Duration of isolation	Standardized isolation duration reported in 40%; most commonly 14 days; 49% stopped after smear conversion; 14% of respondents have no isolation procedure for smear-negative patient with suspected pulmonary TB	Isolation until non-infectious status achieved, based on clinical and microbiological criteria	Airborne isolation recommended until smear conversion	Isolation rarely required in children; Return to school/work after 2 weeks if clinically improving and adherent. Prolonged isolation may be needed in adolescents or extensive pulmonary disease
Isolation facilities	Negative-pressure single rooms used in 53%; standard single rooms in 39%; no isolation in 3%	Airborne infection isolation rooms recommended when available	Negative-pressure rooms recommended	NA

TB, tuberculosis; MDR-TB, multidrug-resistant tuberculosis; DST, drug susceptibility testing; NAAT, nucleic acid amplification test; HIV, human immunodeficiency virus; NA, not applicable.

**Table 4:** Tuberculosis infection and public health.

TB infection management	Survey findings	WHO module 5 (2022)	ERS/ECDC statement (2017)	IJTLD clinical standards (2023)
TB infection testing	50% systematically ask for TST and 55% ask for IGRA	TST or IGRA test may be used, with IGRA preferred in BCG-vaccinated children	TST or IGRA recommended	TST or IGRA recommended
Indications for TB infection treatment	Systematic treatment in 79%; selective strategies based on exposure, age or immunodeficiency also reported	Preventive treatment recommended for children: - aged <5 years who are household contacts of a patient with TB disease, regardless of TB infection testing - aged 5–19 years with confirmed TB infection - any age living with HIV or other immunocompromising conditions	TB infection testing recommended for contacts of infectious TB and other high-risk groups (HIV, anti-TNF, dialysis, transplant candidates, silicosis); if infection identified, exclude active TB and consider preventive treatment	Preventive treatment recommended for all children
TB infection treatment regimens	3HR most frequently used (59%); followed by 6H, 3–4R, 9H	Recommended regimens include rifamycin-based regimens (3HR, 3HP, 4R) and isoniazid for 6 or 9 months, particularly when rifamycin-based regimens cannot be used	Follow WHO recommendations	Follow WHO recommendations
Contact tracing	Standardized procedures reported by 83%; mainly coordinated by public health authorities	Systematic contact investigation recommended, with national TB programs	Public health-led contact tracing emphasized	Public health services

TB, tuberculosis; TST, tuberculin skin test; IGRA, interferon-gamma release assay; HIV, human immunodeficiency virus; TNF, tumor necrosis factor; H, isoniazid; R, rifampicin; HR, isoniazid plus rifampicin; 3HR, 3-month regimen including isoniazid and rifampicin; 3HP, 3-month regimen including isoniazid and rifapentine; 4R, 4-month regimen including rifampicin; 6H, 6-month regimen including isoniazid; 9H, 9-month regimen including isoniazid.

## 3 Results

### 3.1 Participants

**Figure 1:**
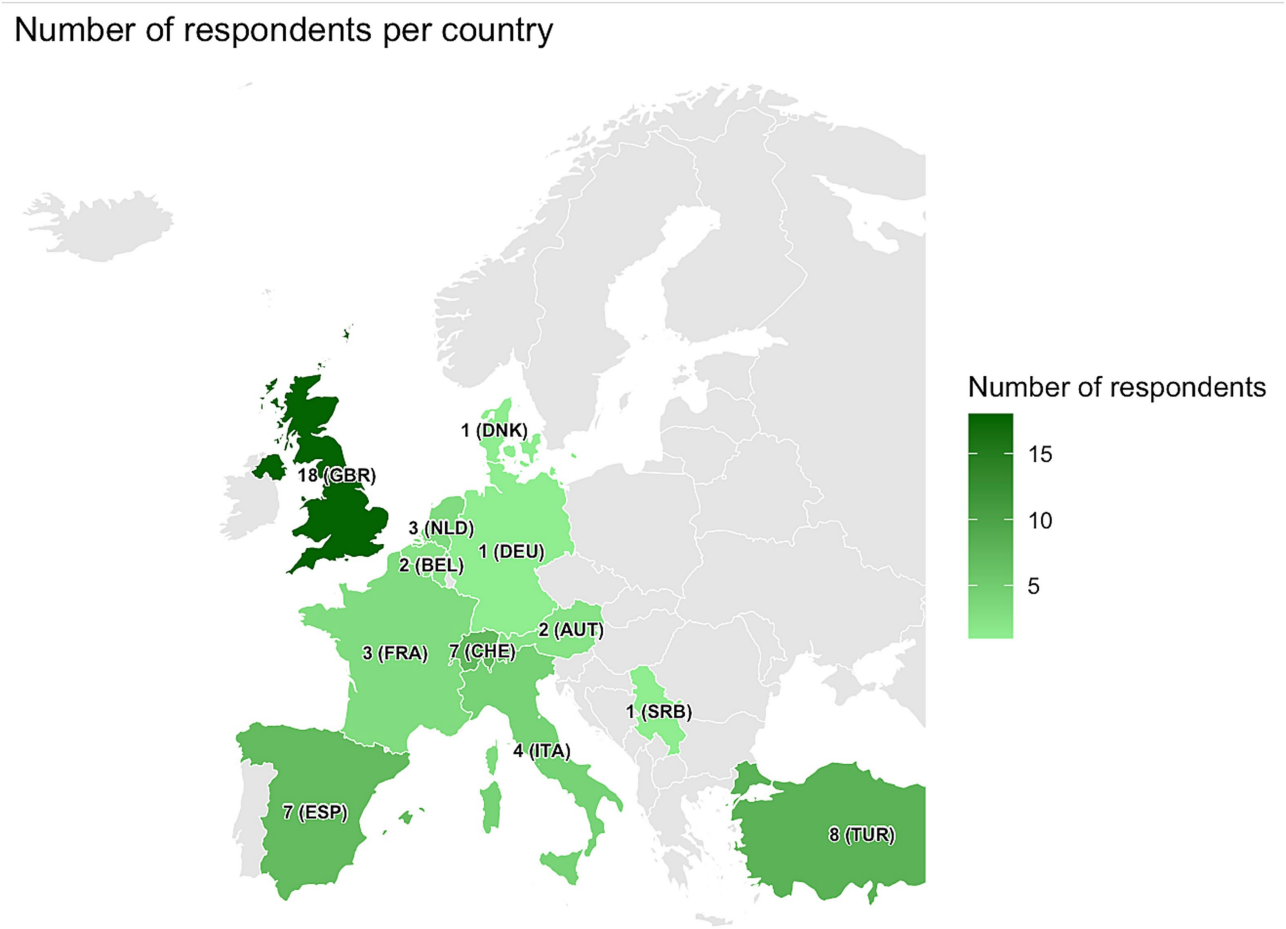
Number of respondents per country. Not on the map: Australia *n* = 1, South Africa *n* = 1, the United States *n* = 1.

A total of 63 respondents completed the questionnaire, with 57 (90%) working in European countries (United Kingdom, Turkey, Spain, Switzerland, Italy, France, the Netherlands, Austria, Belgium, Denmark, Germany, and Serbia) and 6 (10%) working in non-European countries (India, Afghanistan, Australia, South Africa, and the United States of America; Figure 1). Three responses (Afghanistan *n* = 1, India *n* = 2) were excluded from the analysis because these countries are not classified as high-income. Participants are primarily pediatric infectious diseases specialists (40%, 25/60), followed by microbiologists (24%, 15/60), tuberculosis clinical nurse specialists (14%, 9/60), adult infectious diseases specialists (10%, 6/60), pediatricians (6%, 4/60), pediatric respiratory medicine specialists (5%, 3/60), and adult respiratory medicine specialists (2%, 1/60). Respondents follow primarily national recommendations for tuberculosis management (79%, 50/60), approximately half of them also follow WHO recommendations (51%, 32/60), while fewer rely on institutional guidelines (19%, 12/60).

### 3.2 Microbiological TB diagnosis

For children unable to expectorate, nearly half of respondents request an early-morning gastric aspirate as the first-choice sample (47%; 24/51), followed by use of induced sputum or gastric aspirate (29%; 15/51) depending on the age of the child, while induced sputum is requested by 18% (9/51; Table 1). A smear microscopy and culture are systematically performed by 86% (44/51) and 63% (32/51) respondents, respectively. Rapid molecular testing is routinely performed by 80% (41/51) of respondents, whereas 12% (6/51) report using rapid molecular testing only in patients with a positive smear microscopy. Most respondents report being able to perform nucleic acid amplification test (NAAT) on pulmonary samples (84%, 43/51), cerebrospinal fluid (69%; 35/51), and tissue biopsies (59%; 30/51), whereas only a minority indicate the ability to request NAAT on stool specimens (37%; 19/51). Most respondents report performing NAAT on up to three respiratory samples when initial tests were negative (41%; 21/51), while smaller proportions limited testing to one sample (25%; 13/51) or one to two samples if the first was negative (22%; 11/51). Most participants (88%; 45/51) report systematically performing culture when NAAT results are negative. Bronchoscopy is rarely used as a first-line diagnostic procedure in children with suspected pulmonary TB (12%, 6/51) and is performed by 41% of respondents (21/51) when initial microbiological tests are negative, and clinical suspicion remains high. It is also used in cases of suspected drug-resistant TB (2%, 1/51), for radiological findings suggestive of TB such as hilar adenopathy or cavitation (18%, 9/51), and for evaluation of alternative diagnoses (10%, 5/51). Bronchoscopy is not available in 18% of centers (9/51). In cases of strong clinical suspicion of pulmonary tuberculosis with negative microbiological results, including bronchoscopy, most respondents report initiating treatment immediately (73%; 37/51), while fewer prefer to wait for culture confirmation (6%; 3/51) or to decide based on clinical severity (18%; 9/51). On the initial sample, most respondents request molecular testing for drug resistance, with 47% (24/51) asking for rifampicin resistance alone and 41% (21/51) requesting combined rifampicin and isoniazid resistance detection, whereas only a small minority does not request resistance testing (4%; 2/51) or were unsure (8%; 4/51).

Just over a third of participants (35%; 18/51) have access to adenosine deaminase (ADA) test, and it is mainly used on pleural samples (89%; 16/18), cerebrospinal fluid samples (50%; 9/18), or peritoneal fluid (33%; 6/18). Use of lateral flow urine lipoarabinomannan (LF-LAM) assay for diagnosing TB in HIV-positive patients was uncommon: only 3 participants (6%, 3/51) report using this test. The use of interferon-gamma release assays (IGRAs) on non-blood specimens (such as pleural effusion, cerebrospinal fluid or peritoneal fluid) for the diagnosis of active TB is uncommon, with only 8% of respondents (4/51) reporting their use, while the majority does not perform IGRAs on non-blood samples (84%, 43/51) and 8% (4/51) are unsure.

### 3.3 Isolation practice

For patients with smear-positive sputum, 40% of the centers (14/35) report a standardized isolation duration (Table 3). Among these, the majority (86%; 12/14) use 14 days, 8% (1/12) use 21 days, and 8% (1/12) isolate until discharge, if admitted. Otherwise, isolation is stopped after sputum smear negativity in 81% (17/21), after resolution of cough in 10% (2/21), after 14 days if clinical improvement and exclusion of MDR-TB in 5% (1/21) and unspecified in 5% (1/21). For patients with confirmed active pulmonary tuberculosis, 52% of respondents (18/35) use single rooms with negative pressure, 40% (14/35) use classical single rooms, 3% (1/35) have no isolation procedure, and 6% (2/35) are unspecified. For smear-negative patients with suspected pulmonary tuberculosis, 40% (14/35) isolate only hospitalized patients, 31% (11/35) isolate only if clinical or radiological abnormalities are present, 14% (5/35) do not isolate, and 14% (5/35) follow other procedures.

### 3.4 TB treatment

Treatment regimens are mostly standardized. For presumed fully susceptible tuberculosis (excluding TB meningitis or bone disease), 100% of respondents (36/36) use the standard 6-month 2HRZ(E)/4HR regimen (Table 2). Among respondents, 47% (17/36) use the 4-month 2HRZ(E)/2HR regimen for limited disease following Turkova 2022 criteria. For rifampicin dosing, 67% of respondents (24/36) use 10–20 mg/kg, 6% (2/36) use >20 mg/kg, and 28% (10/36) prescribe usually between 10 and 20 mg/kg and adjust to >20 mg/kg depending on conditions such as neurological tuberculosis, HIV co-infection, disseminated disease, low serum levels, or concern for resistance. For TB meningitis, 67% (22/36) of respondents use the standard 2HRZE/10HR regimen, while the remaining participants employ alternative regimens, such as: addition of fluoroquinolone (25%, 9/36), modified standard regimens (ethambutol substitutions with fluoroquinolone or ethionamide; [11%; 4/36]), and a 9-month standard regimen course (2HRZE/7HR; [3%; 1/36]). For TB meningitis, a 4-week corticosteroid course is most commonly used (58%; 21/36), with fewer clinicians opting for 2 weeks (14%; 5/36), 6–8 weeks (17%; 6/36) or 12 weeks (6%; 2/36). Corticosteroids are not used by 2/36 (6%) of respondents. Fluoroquinolones are most commonly prescribed as first-line treatment when isoniazid resistance is suspected (49%; 17/36) and for central nervous system TB (33%; 12/36). Regarding microbiological monitoring during treatment, respondents reported collecting specimens to check for culture conversion at multiple time points: 2 weeks after treatment initiation (56%; 19/34), 1 month (26%; 9/34), 2 months (35%; 12/34), 3 months (6%; 2/34), 4 months (6%; 2/34), at the end of treatment (15%; 5/34), with 15% (5/34) not performing post-treatment culture checks, and 9% (3/34) indicating other timings. Baseline blood testing in asymptomatic patients is routinely performed by most respondents (92%; 33/36). Laboratory toxicity monitoring (full blood count and/or transaminase) is routinely performed by most respondents (67%; 24/36), while smaller proportions restricted monitoring to patients with symptoms of toxicity and/or comorbidities (27%; 10/36). Routine HIV testing for patients diagnosed with active tuberculosis is widely implemented, with 86% (31/36) offering testing to all patients. Pyridoxin is prescribed routinely with isoniazid by 47% (17/36) of respondents, and in specific cases when the patient is malnourished (42%; 15/36), exclusively breastfed (28%; 10/36), or has liver, renal, or HIV-related comorbidities (each 17%; 6/36). During the first 2 months of ethambutol treatment, 61% (22/36) of respondents request an ophthalmology examination. Routine therapeutic drug monitoring of rifampicin and isoniazid is uncommon, with only 8% (3/36) of respondents reporting systematic measurement; most indicated selective testing based on clinical concerns, particularly suspected malabsorption (47%; 17/36) or suspected poor adherence (44%; 16/36), while 25% (9/36) never assessed drug blood levels.

After discharge, directly observed therapy (DOT) is commonly implemented, most often through community nurses or pharmacies (61%; 22/36), or DOT delivered by trained personnel (22%; 8/36), while 17% (6/36) reported not using DOT. Standardized procedures for tracing contacts of tuberculosis patients are reported by most respondents (86%; 30/35). Responsibility for tuberculosis contact tracing is primarily assigned to public health authorities (78%; 28/36), with fewer respondents reporting accountability by other entities, such as hospital infectious disease teams (*n* = 2), TB nursing team (*n* = 2), TB unit (*n* = 2), hospital pulmonary team (*n* = 1).

### 3.5 Tuberculosis infection

Most respondents systematically treat children with tuberculosis infection (79%, 22/28), while few do treat only if children have a history of TB exposure in the past 2 years (18%, 5/28), if they have immunodeficiency (14%, 4/28), or if they are less than 5 years of age (4%, 1/28; Table 4). The most common regimen for treatment of tuberculosis infection is isoniazid and rifampicin for 3 months (59%, 16/27), followed by isoniazid 6 months (37%, 10/27), rifampicin 3–4 months (26%, 7/27), isoniazid 9 months (15%, 4/27), with one respondent using isoniazid and rifapentine 3 months (4%, 1/27).

## 4 Discussion

Overall, the diagnostic and management practices reported in this survey show substantial concordance with international and European recommendations, while also revealing areas of heterogeneity and incomplete implementation. Most respondents report using gastric aspirates or induced sputum as first-line specimens in children unable to expectorate, in line with WHO, ERS/ECDC, and IJTLD guidance, which recognize these samples as central to pediatric TB diagnosis (5–7). A notable gap identified in this survey is the limited use of stool specimens for TB diagnosis, with just over a third of respondents reporting access to NAAT testing on stool, despite growing evidence supporting its diagnostic value in children. Multiple studies and meta-analyses have demonstrated that Xpert MTB/RIF and Xpert Ultra performed on stool achieve moderate sensitivity but excellent specificity, with diagnostic performance comparable to gastric aspirates in some pediatric populations (11–13). Consequently, WHO now supports stool as an alternative specimen for NAAT in children with suspected pulmonary TB when respiratory samples are difficult to obtain, and IJTLD standards similarly recognize stool testing to improve diagnostic yield while reducing reliance on more invasive procedures (5, 7). The low uptake of stool-based NAAT in this survey may be explained by differences in access and laboratory capacity, as well as the relatively recent introduction of this recommendation, which is not included in earlier ERS/ECDC guidelines, rather than by clinician preference or ambiguity in current recommendations. As standardized protocols and automated platforms become more widely implemented, stool-based NAAT is likely to play an increasingly prominent role in pediatric TB diagnostics, particularly for young children and in outpatient or decentralized settings, offering an opportunity to further harmonize clinical practice with evolving international recommendations (5, 11, 14). Bronchoscopy in pediatric tuberculosis is a selective diagnostic and interventional tool, reserved for children with negative initial microbiological tests, high clinical suspicion, or radiological findings suggestive of TB (15–17). Consistent with these recommendations, our survey shows that bronchoscopy is rarely used as a first-line procedure and is generally applied only in complex cases. Bronchoscopy can improve diagnostic yield and guide therapeutic interventions, such as relieving airway obstruction, with low complication rates in specialized centers (15, 18). These findings support a stepwise strategy prioritizing non-invasive sampling while reserving bronchoscopy for selected pediatric TB cases, in line with WHO and observational study (5, 19).

The high uptake of rapid molecular testing as an initial diagnostic tool and the frequent performance of culture following negative NAAT results are consistent with current standards advocating molecular confirmation combined with culture to enable drug susceptibility testing (DST) (5–7). However, a minority of respondents report restricting NAAT use to smear-positive samples, diverging from recommendations that support NAAT irrespective of smear status in children, in whom disease is typically paucibacillary (5, 7). Although NAAT represents the most sensitive diagnostic tool for tuberculosis, practices vary widely, with some respondents limiting testing to one or two samples. In adults, several studies have shown that most of the diagnostic yield is achieved with the first two NAATs, while the incremental value of a third test is limited (20–22). Pediatric-specific data are scarce, but these adult findings raise questions about the optimal number of NAATs in children. Most participants report requesting molecular drug resistance testing on initial samples, in keeping with the strong emphasis on early detection of rifampicin resistance (5–7). However, heterogeneity in the scope of resistance testing, particularly the inconsistent detection of isoniazid resistance, suggests incomplete integration of newer assays such as Xpert MTB/XDR, despite their endorsement in recent WHO and IJTLD standards (5, 7).

Isolation and infection prevention practices show marked heterogeneity across centers. Only half of respondents report a standardized isolation duration for smear-positive pulmonary TB, most commonly 14 days, while others rely on sputum smear conversion or clinical improvement to discontinue isolation. This variability reflects the limited prescriptiveness of current guidance, as WHO and ERS/ECDC recommend discontinuation of isolation based on clinical, microbiological, and treatment adherence criteria rather than a fixed duration (5, 6). The frequent use of negative-pressure rooms for confirmed pulmonary TB aligns with recommendations for airborne infection isolation, particularly for smear-positive cases (5, 6). However, the choice of isolation often reflects both hospital resources and perceived patient contagiousness. The substantial use of conventional single rooms, and the minority reporting no isolation, likely reflects recognition of the generally low infectiousness of young children, as emphasized in the IJTLD Clinical Standards, which state that prolonged isolation is rarely required in pediatric TB except in adolescents or children with extensive pulmonary disease, or when hospital resources are limited (7). The heterogeneous approach to isolating smear-negative suspected cases further underscores the lack of consensus in low-incidence, high-income settings, where infection control must be balanced against resource use and the psychosocial impact of isolation. This variability highlights the need for clearer operational guidance and implementation strategies, such as audit and feedback, to support more consistent application of infection prevention and control measures across centers.

Treatment practices reported in this survey were largely standardized. All respondents reported using the standard 6-month 2HRZ(E)/4HR regimen for presumed fully susceptible TB, consistent with WHO, ERS/ECDC, and IJTLD recommendations (5–7). Less than half of respondents reported using a 4-month regimen for non-severe disease, despite strong evidence from the SHINE trial and subsequent WHO endorsement for eligible children (5, 8, 9). This relatively low uptake of shorter treatment may reflect implementation challenges, including uncertainty around eligibility criteria, delays in national guideline updates, and clinician confidence in applying newer treatment strategies. Fluoroquinolones were frequently incorporated into treatment regimens: nearly half of respondents reported using a fluoroquinolone as first-line therapy when isoniazid resistance was suspected or documented. WHO recommends a six-month regimen including rifampicin, ethambutol, pyrazinamide, and a later-generation fluoroquinolone for children and adolescents with confirmed or suspected isoniazid-resistant, rifampicin-susceptible TB (5). This recommendation was informed by multiple cohort studies and meta-analyses demonstrating improved treatment outcomes with fluoroquinolone-containing regimens compared with regimens relying exclusively on first-line drugs (23–26). A large multicenter cohort study by Min et al. showed significantly higher treatment success rates among patients treated with fluoroquinolone-containing regimens, reinforcing the clinical benefit of this approach (27). These data provide a strong evidence base for the frequent use of fluoroquinolones reported by survey respondents in cases of suspected isoniazid resistance.

In addition to their role in drug-resistant disease, fluoroquinolones are increasingly used in tuberculous meningitis owing to their favorable pharmacokinetic and pharmacodynamic properties. Levofloxacin and moxifloxacin achieve high cerebrospinal fluid concentrations, often exceeding the minimum inhibitory concentration for *Mycobacterium tuberculosis*, in contrast to ethambutol, which has limited penetration across the blood–brain barrier (28, 29). Pharmacokinetic studies and adult tuberculous meningitis trials have demonstrated that higher fluoroquinolone exposure is associated with improved bactericidal activity in the central nervous system, providing a biological rationale for their use in tuberculous meningitis regimens (30, 31). The heterogeneity observed in fluoroquinolone use for tuberculous meningitis in our survey likely reflects ongoing uncertainty regarding optimal regimen composition and duration in children, as well as differences between guideline recommendations. While WHO continues to recommend a standard 12-month regimen for tuberculous meningitis, IJTLD standards acknowledge alternative strategies, including fluoroquinolone substitution, particularly in severe cases (5, 7). Most respondents prescribe adjunctive corticosteroids in tuberculous meningitis; however, a small minority still do not, despite strong evidence from Cochrane reviews demonstrating their benefit in reducing mortality and neurological complications (32).

Use of the 3-month isoniazid and rifapentine regimen for latent TB was very low. While the reasons for this limited uptake were not assessed in the survey, restricted access to rifapentine in some countries may contribute. Surveys of drug availability in Europe have shown that rifapentine is available in only a minority of countries (approximately 14% of the 43 countries surveyed) (33). Addressing barriers to rifapentine availability could help facilitate broader implementation of shorter preventive and treatment regimens in pediatric TB care.

Monitoring practices during treatment are generally aligned with guidelines but showed considerable variability. Most respondents perform baseline laboratory testing and routine laboratory toxicity monitoring, consistent with WHO recommendations, while IJTLD standards support a more selective approach in low-risk children (5, 7). Microbiological monitoring practices vary widely, reflecting both heterogeneity in clinical practice and lack of concordance between guidelines: WHO and IJTLD emphasize clinical monitoring in children and reserve repeat microbiological testing mainly for bacteriologically confirmed or severe disease, while ERS/ECDC recommends systematic smear and culture assessment at the end of the intensive phase for pulmonary tuberculosis (5–7). Routine therapeutic drug monitoring is uncommon, in line with WHO and IJTLD recommendations that do not support systematic use but endorse targeted testing in selected clinical situation, including suspected malabsorption, drug interactions, poor adherence, or suboptimal clinical response (5, 7). While systematic HIV testing is widely implemented, incomplete uptake across centers highlights the need to strengthen universal routine HIV testing for all children with tuberculosis, as consistently recommended across all three guidelines (5–7). Pyridoxine supplementation practices are heterogeneous, with fewer than half of respondents prescribing vitamin B6 routinely with isoniazid, despite WHO and IJTLD recommendations supporting broader use in infants, adolescents, malnourished children, and those with comorbidities or high-dose isoniazid exposure (5, 7).

Finally, given that pediatric TB is often paucibacillary, microbiological confirmation may be difficult to obtain. The widespread initiation of treatment in cases of strong clinical suspicion despite negative microbiological results mirrors all reference standards, which explicitly recognize the acceptability and necessity of clinical diagnosis in pediatric TB due to its often paucibacillary nature (5–7). Post-discharge management and public health measures are largely consistent with recommendations, with most respondents reporting implementation of directly observed therapy and standardized contact tracing coordinated by public health authorities, reflecting established TB control infrastructures in Europe (6, 34).

The TBSure study complements the multicountry survey by Méchaï et al. on adult TB management in high-income countries, allowing a direct comparison of adult and pediatric practices (10). Both studies show overall adherence to international recommendations, alongside persistent heterogeneity in real-world practice.

Unlike adults, where expectorated sputum is central to diagnosis, pediatric TB relies more on gastric aspirates, induced sputum and stool samples, with less frequent bronchoscopy. NAAT use is higher in children than in adults (80% vs. 64%), though some clinicians in both populations still restrict it to smear-positive cases. Treatment regimens are more standardized in children, with all using first-line therapy and nearly half adopting shorter courses for non-severe TB. Steroid use for central nervous system TB is also more systematic in children, in line with current evidence.

Monitoring and adjunctive care remain more variable in pediatric TB. Culture follow-up spans multiple time points (2 weeks–end of treatment) versus adults who repeat sputum at 2 and 6 months. While baseline laboratory monitoring and HIV testing are widely implemented, pediatric practices are less uniform for pyridoxine supplementation (47% [17/36] vs. 89% [57/63] in adults). Ophthalmological assessment is more systematic in children (60% [21/35]) compared to adult population (50% [32/62]). Therapeutic drug monitoring is performed routinely by 8% (3/36) of respondents and in selected children by 58% (21/36) of respondents whereas in the adult population, therapeutic drug monitoring was performed systematically by 17% (11/64) of respondent and selectively by 23% (15/64). These findings suggest that pediatric TB management relies more on individualized, case-by-case decisions, reflecting limited pediatric-specific evidence, guideline variability, and differences in national policies.

This study provides one of the few multi-country overviews of pediatric tuberculosis management practices in high-income settings, addressing an important gap in the literature dominated by adult data. The use of a standardized survey distributed through a European professional network enabled comparison of real-world practices across multiple countries and key domains of pediatric TB care, including diagnosis, treatment, infection control, and monitoring. The results of our study reflect a hospital-based practice. Although this setting may introduce a potential bias, we believe the risk is minimal, as it is highly unlikely that children with TB in high-income countries would be managed exclusively by private pediatricians, given the diagnostic challenges and the higher risk of severe disease, particularly in infants.

Beyond tuberculosis, these findings align with broader evidence that guideline implementation, standardization of care, and the ability to adapt care pathways in response to evolving evidence are key determinants of health system resilience (35). Monitoring practice variation through transparent benchmarks can help identify gaps and prioritize actions to improve both performance and equity in real-world settings.

Limitations include the voluntary and self-reported nature of the survey, which may introduce selection and reporting biases. As participation largely involved professionals affiliated with networks with expertise in mycobacterial infections, the results may over-represent practices from specialized centers and may not fully reflect national practice patterns. The uneven country representation, with most participants working in Western Europe and Turkey, where diagnostic tools may be more accessible than in other parts of Europe, limits generalizability in Europe and beyond high-income settings. In addition, evolving guidelines and diagnostic tools may have influenced practices during the study period. The ERS/ECDC recommendations precede those from the WHO and IJTLD, which may explain some of the differences between these guidelines. Finally, the survey did not include data on patient outcomes or treatment success and was therefore not designed to evaluate the clinical consequences of practice heterogeneity.

## 5 Conclusion

Taken together, these findings suggest that while core principles of pediatric TB diagnosis and management are well integrated into practice in high-income European settings, meaningful variability persists in the adoption of emerging diagnostics, infection control measures, adjunctive therapies, and monitoring strategies. While these differences may partly stem from disparities in access to specific management tools across settings, targeted initiatives to harmonize national guidelines and promote the adoption of updated international recommendations have the potential to further enhance pediatric TB care across Europe.

## Data Availability

The raw data supporting the conclusions of this article will be made available by the authors upon request and after a clarification of the ethical responsibility.
