# Supplementary material for: TBSure: an ESGMYC survey of pediatric TB management practices in high-income countries
Source: Front Public Health. 2026 May 19;14:1829612. doi: 10.3389/fpubh.2026.1829612 (PMC13226504; doi:10.3389/fpubh.2026.1829612)
Supplement: Supplementary file 1 [file Supplementary_file_1.docx]

Supplementary Material

# Supplementary Data

**TBsure paediatric study - questionnaire**

General information

**Prerequisite: Management of a proven/suspected case of tuberculosis disease in a patient under 18 years of age in the last 2 years**

1. Reporting country

2. Reporting centre

3. Profession

Infectious disease specialist (paediatric)

Infectious disease specialist (adult)

Microbiologist

Paediatrician

Respiratory medicine specialist (paediatric)

Respiratory medicine specialist (adult)

Other (please specify)

4. How many case of active tuberculosis (adult patient) do you diagnose each year (on average)?

< 5 cases/ year

5-10 cases/ year

> 10 cases / year

5. How many case of active tuberculosis (paediatric patient) do you diagnose each year (on average)?

< 5 cases/ year

5-10 cases/ year

> 10 cases / year

6. What recommendations for the management of tuberculosis do you follow?

Institutional recommendations

National recommendations

WHO recommendations

Other (please specify)

TBsure paediatric study

Screening test for latent tuberculosis infection

7. When do you perform tuberculin skin test (TST)?

For all children

Only for children aged less than 5 years

Only for children aged less than 2 years

You never request TST

Other (please specify)

8. When do you perform interferon-gamma release assays (IGRA)?

For all children

Only for children aged more than 5 years

Only for children aged more than 2 years

For all children with a history of BCG vaccination

You never request IGRA

Other (please specify)

9. If you use TST , which induration size do you consider positive (patient WITHOUT a

history of BCG vaccination)?

>/= 5 mm

>/= 10 mm

>/= 15 mm

>/= 20 mm

Other (please specify)

10. If you use TST , which induration size do you consider positive (patient WITH a history of BCG vaccination)?

>/= 5 mm

>/= 10 mm

>/= 15 mm

>/= 20 mm

Other (please specify)

Diagnosis approaches for active tuberculosis

11. What type of diagnostic tests are available in your clinical setting?

Smear microscopy

Rapid PCR tests (i.e Xpert MTB/RIF , Xpert MTB/RIF Ultra)

Moderate complexity automated nucleic acid amplification tests (NAATs) (i.e in-house real-time PCR)

Loop-mediated isothermal amplification

Urine antigen test (lateral flow urine lipoarabinomannan (LF-LAM) assay)

Culture

I don't know

Other (please specify)

12. What is your initial diagnostic test for patients with signs and symptoms of pulmonary TB?

smear microscopy

nucleic acid amplification test (NAAT)

culture

urine antigen test (lateral flow urine lipoarabinomannan assay)

unknown

Other (please specify)

13. When do you perform a NAAT?

For all patient with suspected pulmonary TB

For patients with a positive smear microscopy

Other (please specify)

14. On what type of samples can you carry out a NAAT?

Pulmonary samples (including bronchoscopy)

Nasopharyngeal aspirate

Stool

Urine

Cerebrospinal fluid

Tissue biopsy

NAAT not available

Another specimen (please specify)

15. What is the first-choice sample you ask for in cases of suspected active pulmonary tuberculosis for a child who is able to expectorate?

Spontaneous expectorated sputum

Induced sputum

Early morning gastric aspirate

Nasopharyngeal aspirate

Bronchoscopy

Stool

Urine

String test

Another specimen (please specify)

16. What is the first-choice sample you ask for in cases of suspected active pulmonary

tuberculosis for a child who is unable to expectorate

Induced sputum

Early morning gastric aspirate

Induced sputum or early morning aspirate depending on the age of the patient

Nasopharyngeal aspirate

Bronchoscopy

Stool

Urine

String test

Another specimen (please specify)

17. When do you perform bronchoscopy in your clinical setting?

As a first-line procedure in case of suspicion of pulmonary TB

Only in case of smear-negative sputum

Not available

On clinical/radiological criteria (please specify)

18. On how many respiratory samples do you perform NAAT?

Only on one sample

Up to 2 samples if the first sample is negative

Up to 3 samples if the first 2 samples are negative

On every sample you have if the previous samples are negative

19. Do you always perform culture if NAAT are negative?

Yes

No

20. In case of strong suspicion of pulmonary TB with NEGATIVE microbiological samples

(for example NAAT negative on every sample, including bronchoscopy), do you:

Start the treatment

Wait for the culture results before starting the treatment

Depending of clinical severity

Other (please specify)

21. Do you use adenosine desaminase (ADA) test?

Yes

No

22. If yes, on wich sample?

pleural eﬀusions

cerebrospinal fluid

peritoneal punction

nodes punction

Other (please specify)

23. Do you use lateral flow urine lipoarabinomannan (LF-LAM) assay to assist in the

diagnosis of TB disease in HIV-positive patients?

Yes

No

I don't know

24. Do you perform IGRAs on non-blood specimens (pleural eﬀusions, cerebrospinal fluid,

peritoneal punction…) for active TB diagnosis?

Yes

No

I don't know

Drug-resistance detection

25. On your initial sample, do you systematically ask for:

Molecular detection of rifampicin resistance

Molecular detection of rifampicin and isoniazid resistance

You do not ask for molecular detection of resistance

Unknown

Other (please specify)

Tuberculosis management

**To be completed by physicians only**

26. Do you prescribe antituberculosis drugs?

Yes

No

27. At what time do you collect sample(s) to check for culture negative conversion for M.

tuberculosis?

2 weeks after the start of treatment

1 month after the start of treatment

2 months after the start of treatment

3 months after the start of treatment

4 months after the start of treatment

At the end of treatment

You do not check culture after the start of treatment

Other (please specify)

28. For patients with smear-positive sputum, do you have a standardized duration of isolation?

Yes

No

29. If yes, how long is this duration?

14 days

21 days

Other (please specify)

30. If you do not have a standardized duration of isolation, in what circumstances do you stop isolation?

Only when you know that the sputum is smear-negative

After resolution of the cough

When you have excluded any drug resistance

Other (please specify)

31. In what kind of isolation room do you hospitalize patients with confirmed active pulmonary tuberculosis?

Classical single room

Single room with negative pressure

No isolation procedure

Other (please specify)

32. What type of respiratory isolation do you apply in case of smear-negative patients with

suspicion of pulmonary tuberculosis?

Isolation only for hospitalized patients

Isolation only if there are abnormalities on clinical/radiological exam

No isolation

Other (please specify)

33. What is your empiric treatment of a presumed fully susceptible tuberculosis (excluding tuberculous meningitis or bone disease)?

2HRZ(E)/4HR (2 months of isoniazid, rifampicin, pyrazinamide, +/- ethambutol then 4 months of

isoniazid + rifampicin)

Other (please specify)

34. Do you use a 4-month treatment regimen (2HRZ(E)/2HR) for patient aged 3 months to 16 years of age?

No

Yes, according to the criteria from Turkova NEJM 2022 (single lobe, no cavity , no bronchial obstruction, no pleural involvement, no miliary disease OR isolated lymph node tuberculosis AND smear negative)

Yes according to other criteria (please specify)

35. What dosage do you use for Rifampicin?

10-20 mg/kg

> 20 mg/kg

10-20 mg/kg usually and >20 mg/kg under certain condition (specify those conditions)

36. What is your standard treatment of tuberculous meningitis?

2HRZE/10HR (2 months of isoniazid, rifampicin, pyrazinamide, ethambutol then 10 months of isoniazid + rifampicin)

Other (please specify)

37. How long do you use corticosteroids for tuberculous meningitis (without taking into account the tap-oﬀ period after corticosteroids use)?

2 weeks

4 weeks

6 weeks

8 weeks

10 weeks

12 weeks

> 12 weeks

Not used

38. When do you prescribe fluoroquinolones as first line treatment?

Never

Suspicion of isoniazid resistance

Osteoarticular tuberculosis

Central nervous system tuberculosis

Other (please specify)

39. Under which conditions do you prescribe vitamin B6 with isoniazid treatment?

Always

Never

if the patient is malnourished

if the patient is exclusively breastfed

if the patient has chronic liver disease

if the patient has renal failure

if the patient has symptomatic HIV infection

if the patient has seizure disorder

Other (please specify)

40. Do you ask for an ophthalmology exam for patient taking ethambutol treatment during the first 2 months of treatment?

Yes

No

41. Do you check drug blood levels following treatment with rifampicin and isoniazid?

Always

Never

If HIV co-infection

If renal failure

If overweighed

If concerned about malabsorption

If concerned about adherence

Unavailable in your clinical setting

Other criteria (please specify)

42. Do you do baseline blood tests (full blood count and/or transaminases) in asymptomatic

patients?

Yes

No

43. For which patients do you monitor biology toxicity after the start of the treatment

None

All patients

Patients with symptoms of toxicity

patients with symptomes of toxicity AND comorbidities

Patients with comorbidities (please specify)

44. Do you regularly monitor biology toxicity (full blood count and/or transaminases) in

asymptomatic patients after the start of the treatment?

No

Yes, every month

Yes, at another timing (specify)

45. Do you oﬀer routine HIV testing for all patients with a diagnosis of active TB?

No

Yes, to all patients

Yes, according to specific criteria (please specify)

46. What are your DOT (directly observed therapy )-implementation procedures?

DOT with community nurses or pharmacy

DOT through trained people

No DOT

47. Do you have standardized procedure for tracing contacts of TB patients?

Yes

No

I don’t know

48. Who is accountable for contact tracing?

Public Health

Hospital infectious disease team

Hospital infection control service

Other (please specify)

49. Do you systematically treat children with latent tuberculosis?

Yes

No

Under condition

50. Under which condition do you treat children with latent tuberculosis?

Children with a history of tuberculosis exposure in the 2 years preceding the diagnosis of latent

tuberculosis

Children with immunodeficiency

According to age (please specify age)

51. For patients with latent tuberculosis, which treatment regimen do you usually use?

Isoniazid 6 months

Isoniazid 9 months

Rifampicin 3-4 months

Isoniazid + rifampicin 3 months

Isoniazid + rifapentine 3 months

Isoniazid + rifapentine 1 month

Other (please specify)
